# Beyond the type 1 pattern: comprehensive risk stratification in Brugada syndrome

**DOI:** 10.1007/s10840-025-02101-z

**Published:** 2025-08-06

**Authors:** Kwan Yau Kan, Aléchia Van Wyk, Toby Paterson, Naveen Ninan, Pawel Lysyganicz, Ishika Tyagi, Ravisankar Bhasi Lizi, Fayza Boukrid, Maha Alfaifi, Alka Mishra, Sai Vamshi Krishna Katraj, Vivetha Pooranachandran

**Affiliations:** 1 Department of Natural Sciences, Middlesex University, The Burroughs, London, NW4 4BT UK; 2 Warwick Medical School, The University of Warwick, Coventry, UK; 3 St George’s University Hospitals, London, UK; 4 North West Anglia Hospitals, Peterborough, UK

**Keywords:** Brugada syndrome, BrS, ventricular arrhythmia, VA, sudden cardiac death, SCD, implantable cardioverter defibrillator, ICD

## Abstract

Brugada Syndrome (BrS) is an inherited cardiac ion channelopathy associated with an elevated risk of sudden cardiac death, particularly due to ventricular arrhythmias in structurally normal hearts. Affecting approximately 1 in 2,000 individuals, BrS is most prevalent among middle-aged males of Asian descent. Although diagnosis is based on the presence of a Type 1 electrocardiographic (ECG) pattern, either spontaneous or induced, accurately stratifying risk in asymptomatic and borderline patients remains a major clinical challenge. This review explores current and emerging approaches to BrS risk stratification, focusing on electrocardiographic, electrophysiological, imaging, and computational markers. Non-invasive ECG indicators such as the β-angle, fragmented QRS, S wave in lead I, early repolarisation, aVR sign, and transmural dispersion of repolarisation have demonstrated predictive value for arrhythmic events. Adjunctive tools like signal-averaged ECG, Holter monitoring, and exercise stress testing enhance diagnostic yield by capturing dynamic electrophysiological changes. In parallel, imaging modalities, particularly speckle-tracking echocardiography and cardiac magnetic resonance have revealed subclinical structural abnormalities in the right ventricular outflow tract and atria, challenging the paradigm of BrS as a purely electrical disorder. Invasive electrophysiological studies and substrate mapping have further clarified the anatomical basis of arrhythmogenesis, while risk scoring systems (e.g., Sieira, BRUGADA-RISK, PAT) and machine learning models offer new avenues for personalised risk assessment. Together, these advances underscore the importance of an integrated, multimodal approach to BrS risk stratification. Optimising these strategies is essential to guide implantable cardioverter-defibrillator decisions and improve outcomes in patients vulnerable to life-threatening arrhythmias.

**Supplementary Information:**

The online version contains supplementary material available at 10.1007/s10840-025-02101-z.

## Introduction

Brugada syndrome (BrS) is an inherited genetic condition characterised by its autosomal dominant pattern. Although BrS is relatively rare, with a prevalence of approximately 1 in 2,000 individuals, it is significant in the context of sudden cardiac death (SCD), contributing to about 4% of such cases [1]. In various studies, it has been observed that around 80% of individuals affected by the condition are male, with symptom onset generally occurring at an average age of 40 years [2, 3].

BrS is diagnosed in individuals without underlying heart disease who present a spontaneous type 1 ECG pattern, regardless of the presence or absence of symptoms. This diagnosis is due to the syndrome’s rarity in the general population and its association with inherent risks. The type 1 pattern can also be provoked by administering a sodium channel-blocking agent as part of a diagnostic test for patients suspected of having concealed BrS, even if they do not exhibit a spontaneous type 1 pattern. However, it is important to note that the provocation induced by medication or fever is less specific than previously believed, as evidence shows that an ECG change can occur in up to 4% of healthy individuals [1]. Current guidelines suggest that an induced type 1 pattern alone requires additional clinical features, such as documented polymorphic ventricular tachycardia (PVT), ventricular fibrillation (VF), arrhythmic syncope, or significant family history to confirm the diagnosis [4].

Traditional risk models based on syncope and spontaneous Type 1 patterns have limited sensitivity in asymptomatic patients. Over the past 30 years since BrS was first described, multiple studies have explored whether additional markers, beyond the spontaneous type 1 pattern, can predict the risk of arrhythmias. Recent electrophysiological studies have revealed an association between BrS ventricular arrhythmias (VA) and abnormalities in the epicardial tissue of the ventricles. Findings demonstrate that factors such as delayed electrical activity in the right ventricular outflow tract (RVOT), the presence of fibrosis, and subtle alterations in both the function and morphology of the heart contribute to the development of VAs [5]. In this review, we aim to investigate the invasive and non-invasive markers and their role in predicting arrhythmic risk.

### Types of ECG pattern

**Fig. 1:**
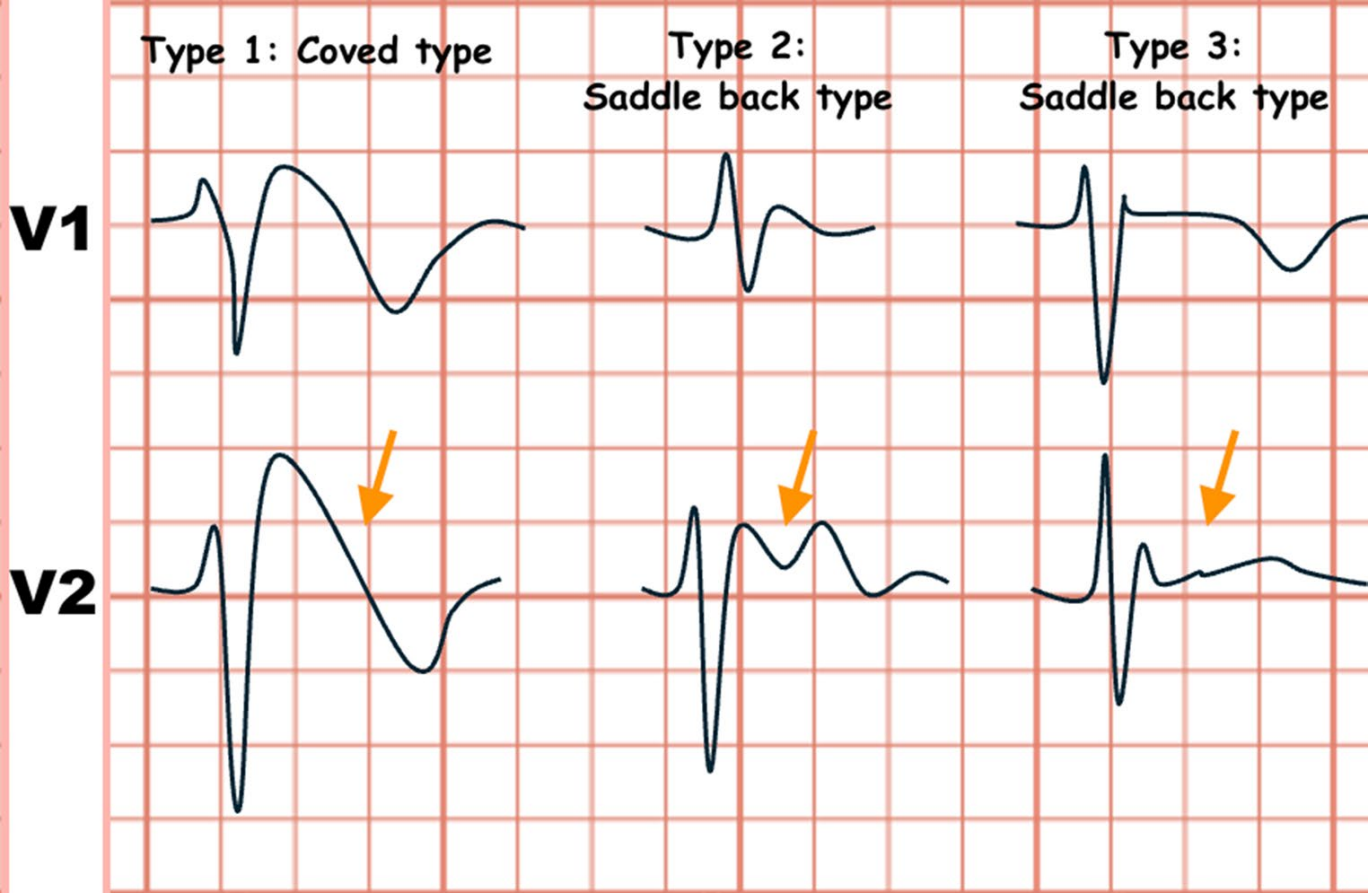
Brugada Syndrome; Types of ECG Pattern

Three specific ECG patterns associated with BrS have been identified (Fig. 1). Out of these, only patients exhibiting Type 1 ECG can be unequivocally confirmed as having BrS, and this type is linked with an increased risk of SCD, with a recent meta-analysis demonstrating a hazard ratio of 2.05 for major arrhythmic events [MAEs] [6]. Type 1 BrS can fluctuate over time, responding to external influences such as fever, changes in vagal tone, and physical exertion. Nonetheless, supportive evidence from electrophysiological studies indicates that approximately 50% of patients with a history of cardiac arrest do not display Type 1 during monitoring [7]. Consequently, the risk of VAs in patients who present with spontaneous Type 2 or Type 3 ECG should not be underestimated, especially for those who have experienced symptoms like syncope.

In the standard ECG configuration, repolarisation patterns induced by BrS are typically identified in at least two of the precordial leads, notably between V1 and V3 [8]. The BrS ECG pattern is predominantly observed when the electrode is positioned over the RVOT, as it is the origin of the abnormal electrical activity associated with BrS [9]. Previous studies have demonstrated that BrS type 1 detection can be increased by the precise identification of the RVOT’s location. To enhance the diagnostic sensitivity for detecting BrS, there is now a recommendation to reposition the precordial leads V1 and V2 to the second or third intercostal space [10]. This adjustment is referred to as the High-Lead ECG. Studies have demonstrated a notable increase in the detection power of Type 1 BrS patterns, with a sensitivity enhancement of up to 30% when electrodes are placed in the second or third intercostal space [11–13].

### Pharmacological provocation testing in Brugada Syndrome

BrS Type 1 may exhibit ‘hidden’ or ‘concealed’ characteristics that can evolve over time, and healthcare professionals are encouraged to conduct comprehensive assessments to determine the presence of BrS Type 1 through provocative testing.

Provocative tests typically involve the intravenous administration of sodium-channel blockers, which include drugs like ajmaline, flecainide, procainamide, and pilsicainide [14]. These medications differ in their ability to inhibit both inward and outward sodium currents in cardiac cells, each possessing unique potencies [14]. The provocative test is particularly valuable for patients exhibiting RVOT abnormalities, especially in cases where spontaneous BrS Type 1 is not observed, or in instances of Saddleback type patterns. In such cases, BrS Type 1 may be unmasked during the testing process. Furthermore, the provocative test serves a critical role in differentiating true BrS from its phenocopy, which can present similarly in diagnostic scenarios [15]. However, the effectiveness and reliability of this test remain subjects of ongoing debate. For instance, a study by Hisamatsu et al., reported a diagnostic prediction rate of 76.4% for identifying BrS in individuals classified as having BrS Type 2 and Type 3 during provocative testing [11]. Conversely, research conducted by Wolpert et al., indicated that the positive predictive value for successfully unmasking BrS from silent gene carriers using either flecainide or ajmaline was merely 35% [16]. A recent review conducted by Wilde et al., evaluating the results of previous provocative test studies on BrS concluded that the sensitivity is good in detecting true negative BrS by using ajmaline. However, its specificity for confirming true BrS diagnosis remains suboptimal [14].

Considering patient safety, it is paramount to acknowledge that the provocative test can induce potentially serious arrhythmias, including ventricular ectopics, non-sustained VT, and VF [14]. While the provocative test has the potential to unveil concealed manifestations of BrS, it is also essential to consider contradictory findings. For example, Ensam et al., and recently, Behr, et al., illustrated that the BrS Type 1 phenocopy could be induced even in otherwise healthy individuals following ajmaline administration, raising further questions about the sensitivity and specificity of this testing method [17, 18].

### β-Angle, duration of the base of the triangle, base at isoelectric line, and base/height ratio

**Fig. 2:**
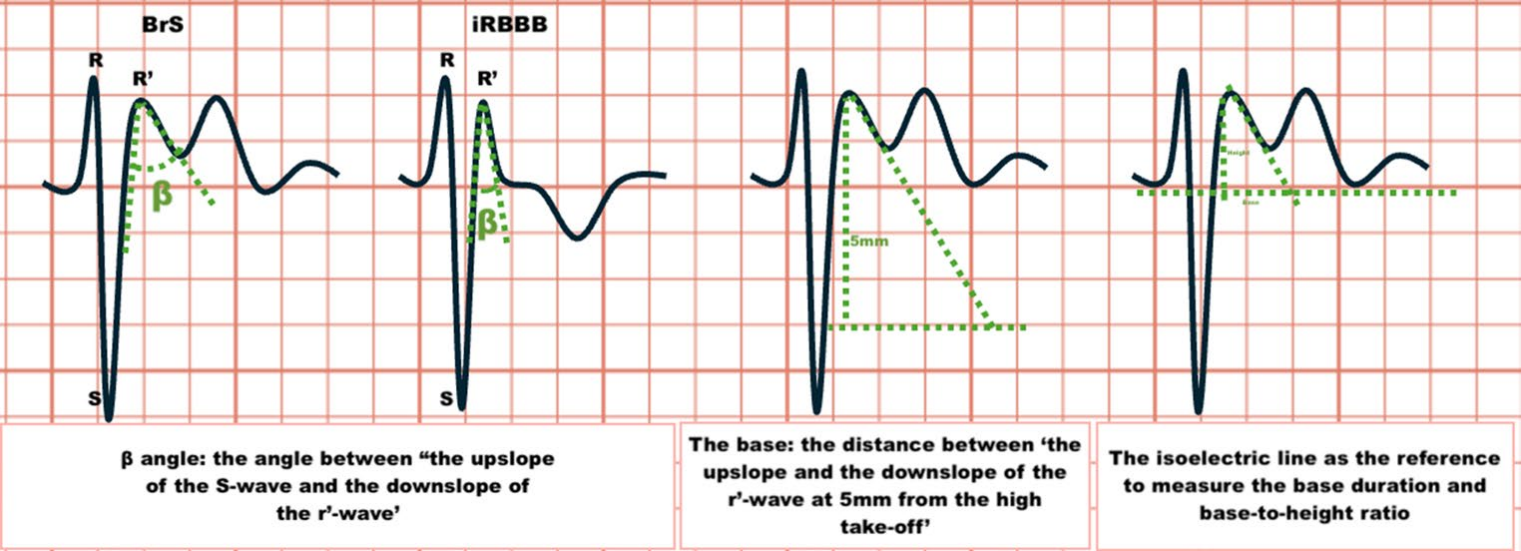
Method to identify BrS Saddleback on an ECG. BrS; Brugada Syndrome, iRBBB; Incomplete right bundle branch block

β-angle, duration of the base of the triangle, base at the isoelectric line, and base/height ratio are electrocardiographic markers that have been proposed as promising adjunctive markers for the diagnosis of BrS, particularly in patients with non-diagnostic ECGs or borderline Brugada patterns (Fig. 2).

The β-angle, defined as the angle between the ascending limb of the S wave and the descending limb of the r′ wave in leads V1 or V2 has been suggested as a morphological ECG feature to help differentiate BrS from Brugada phenocopy (BrP) and other conduction disorders like incomplete right bundle branch block (iRBBB). In BrS patients, a widened β-angle is thought to reflect delayed conduction in the RVOT, a central feature in BrS pathophysiology. Chevallier et al., first proposed that a β-angle ≥ 58° could effectively differentiate BrS type 1 from other causes of ST elevation in the right precordial leads [19]. This concept was later supported by numerous studies as demonstrated by Van der Ree, et al. [20].

Electrophysiologically, this broader angle is attributed to delayed terminal conduction in the RVOT. In BrS patients, the electrical activation progresses more gradually due to slowed right ventricular depolarisation, resulting in a less steep upslope of the S wave and a slower descent from the r′ to the J-point. This results in a wider angle and more flattened terminal portion of the QRS complex. Unlike in right bundle branch block, where conduction delay occurs more proximally, the delay in BrS is localised later in the RVOT, leading to distinct ECG characteristics [20]. The J-point elevation observed in BrS is believed to reflect excitation failure in this region [21].

A 2022 meta-analysis found that the β-angle measured at the fourth intercostal space (IVic) demonstrated a relatively good diagnostic accuracy in patients with suspected BrS based on clinical or ECG features (e.g., type 2 Brugada pattern, unexplained syncope, or family history of SCD), potentially reducing the need for sodium channel blocker provocation testing [22]. However, this finding is based on a small number of studies with limited external validation, and the β-angle has not yet been incorporated into any official risk stratification algorithms.

The base at the isoelectric line, representing the horizontal width of ST elevation at the baseline, the duration of the base of the triangle at 5 mm from the r’-wave, and the base/height ratio (base width divided by the r′ wave height) have similarly been explored as ECG measurements to aid in the diagnosis of BrS [23]. These parameters may help differentiate BrS from BrP, where ECG patterns appear similar but resolve with correction of an underlying, reversible condition such as myocardial ischaemia, electrolyte imbalance, or drug effects [15].

### Signal average electrocardiogram

Signal-averaged electrocardiography (SAECG) is a non-invasive technique that enhances the detection of low-amplitude, high-frequency signals, termed late potentials at the terminal portion of the QRS complex. These late potentials (LPs) reflect delayed conduction in the myocardium and have been proposed as surrogate markers of arrhythmogenic substrates in various cardiac disorders, including BrS [24]. Given the propensity for VAs to originate in structurally and electrically abnormal areas, SAECG has been investigated as a potential tool for risk stratification in BrS. A standard LP criteria was developed in 1994, followed by a modified criteria suggested by the Task Force Committee of the European Society of Cardiology, American Heart Association, and American College of Cardiology in 2010 [24, 25]. An SAECG was considered abnormal when any one of the three following criteria in the absence of a QRS duration of ≥ 110 ms on the standard ECG was met: (1) filtered QRS > 114 ms; (2) root-mean-square voltage < 20 uV in the terminal 40 ms; and (3) a voltage < 40 uV for more than 38 ms (Table 1).

The relevance of SAECG in BrS stems from the syndrome’s characteristic conduction disturbances, particularly in the RVOT. The presence of abnormal LPs are thought to correlate with areas of slowed conduction and delayed depolarisation. Multiple studies have demonstrated that BrS patients with abnormal SAECG parameters are at increased risk of life-threatening arrhythmic events [26, 27].

A prospective single-centre study conducted between 2003 and 2021 assessed SAECG parameters in 117 patients with BrS to evaluate their prognostic significance. The study focused on filtered QRS duration, root mean square voltage of the terminal 40 ms (RMS40), and the duration of low-amplitude signals (< 40 µV) in the terminal portion of the QRS (LAS40), recorded in both conventional and modified right precordial leads. During a median follow-up of 4.1 years, 6.8% of patients experienced MAEs, including SCD and appropriate ICD shocks. The risk of events increased linearly with prolonged filtered QRS, and a risk score incorporating filtered QRS ≥ 113 ms and RMS40 < 13 µV was significantly associated with MAEs. Patients with both abnormal markers showed a sevenfold higher risk of events (HR 7.17, *P* = 0.025), independent of symptomatic status or ECG pattern [26]. Subsequent work has attempted to refine the prognostic utility of SAECG. Takahashi et al., reported that abnormal SAECG findings on a novel unipolar Holter system were associated with sustained VA or SCD [27].

Additional evidence for the pathophysiological relevance of SAECG in BrS is provided by Ciconte et al., who prospectively studied 250 patients undergoing both SAECG and epicardial mapping. They found that LPs detected non-invasively were significantly associated with larger arrhythmogenic substrates (AS) and longer epicardial delayed potentials. Notably, LPs were present in 85.4% of symptomatic BrS patients compared to only 20.1% of asymptomatic individuals, and their presence correlated with spontaneous Type 1 ECG patterns, SCN5A mutations, and larger AS areas. An AS size ≥ 3.5 cm² identified LPs with high accuracy (AUC 0.88, sensitivity 86%, specificity 88%). These findings suggest that SAECG abnormalities reflect underlying epicardial conduction delay [7].

However, despite promising early results, the clinical utility of SAECG in routine risk stratification for BrS remains debated. The methodological variability in SAECG acquisition and interpretation limits its application. Parameters such as signal filtering, noise threshold, and QRS onset/offset determination can vary between institutions and influence outcomes. Furthermore, interobserver variability in interpreting SAECG results complicates comparisons across studies. Nevertheless, there is increasing interest in incorporating SAECG into multiparametric risk models that combine clinical, genetic, and electrophysiological data. To advance this integration and optimise the identification of BrS patients who may benefit from ICDs, large cohort studies employing standardised, multi-marker approaches are urgently needed [28].

**Table 1:** Standard and modified criteria for identifying late ventricular potentials

	Standard criteria ​​( McKenna et al. 1994 [24]	Modified criteria ​​( Marcus et al. 2010 )​ [25]
Filtered QRS duration	> 120 ms	> 114ms
The duration of the terminal part of the QRS complex with amplitude	> 40 µV	<40 µV for > 38ms
The root mean square signal amplitude	Last 30ms of the signal ≤20 µV	Last 40ms of the signal ≤20 µV

### Fragmented QRS complex

**Fig. 3:**
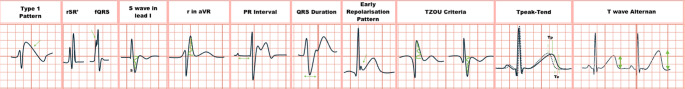
ECG Risk Markers

Fragmented QRS (fQRS) is a simple, non-invasive ECG marker of depolarisation abnormalities. It is defined by the presence of an additional R wave (R’), notching in the nadir of the S wave, more than one R’ wave in two contiguous leads, or more than two notches in the R or S waves in two consecutive leads in the presence of bundle branch block (Fig. 3) [29]. fQRS is a known marker of myocardial injury and conduction delay. Since BrS has been linked to increased collagen and fibrosis in the RVOT, fQRS in BrS may likely indicate conduction delay in the right ventricle. Morita et al., previously demonstrated this using an isolated canine right ventricular tissue model of BrS and demonstrated that epicardial activation delay led to the reproduction of fQRS in the transmural ECG [30].

The use of fQRS in assessing arrhythmic risk has been investigated in several studies. According to findings from Priori et al., BrS patients exhibiting fQRS in leads V1 to V3 were nearly four times more likely to encounter future arrhythmic events [31]. Similarly, a 2017 meta-analysis including 1,637 BrS patients (mean age: 47 ± 11 years) found that fQRS was an independent predictor of future arrhythmic events (relative risk (RR): 3.88, 95% CI: 2.26–6.65, *p* < 0.00001). When VF was analysed as an independent endpoint, the RR was 3.61 (95% CI: 2.11–6.18, *p* < 0.00001), suggestive of fQRS as a valid arrhythmogenic risk marker in BrS [32]. Morita, et al., further demonstrated that fQRS in the inferior, lateral, and RVOT in BrS were all associated with VF events (HR 1.2 to 8.5) [33]. Similarly, Yonezu, et al., conducted a retrospective analysis in early repolarisation syndrome patients and demonstrated a significantly higher presence of fQRS in patients with recurrent VF (*n* = 5, 100%) compared to those with non-recurrent VF (*n* = 10, 10%), *P* = 0.002 [34]. Despite this significant risk association, the use of fQRS as a parameter for risk stratification in BrS has not gained widespread adoption. This hesitancy may stem from the variance in definitions and methodologies surrounding fQRS utilised across different studies (Table 2) [35].

**Table 2:** Various definitions of fQRS in studies assessing risk stratification for sudden cardiac death

Authors	Definition of fQRS
​​Take et al. [36]	Average number of spikes present in V1-V3 = 7 ± 2
​​Morita et al. [30] Maury et al. [37]	Presence of ≥ 4 spikes in the QRS in V1/V2/V3 or ≥ 8 spikes in V1- V3
​​Priori et al. [31]	More than 2 spikes present in V1/V2/V3

### Early repolarisation pattern

Early repolarisation (ER) is an electrophysiological abnormality identifiable using the 12-lead ECG led by two competing mechanism theories. The historical, widely accepted theory describes ER to be a result of a repolarisation abnormality, with differences in the prominence of the phase 1 notch of non-nodal myocyte action potential cycles within different regions of the left ventricle. Expressly, it is noted that a more prominent phase 1 notch in the epicardium and lesser so in the endocardium gives rise to a transmural voltage gradient. These changes in action potential repolarisation heterogeneity are believed to be directly caused by abnormalities in transient outward K + current (I_to_) [38]. The second, less popular theory is that ER is a BrS parallel due to overlaps in the two conditions’ manifestations, affecting the left ventricle (LV) rather than the right ventricle (RV), as observed in BrS. This theory of mechanism proposes that ER is a result of a depolarisation abnormality and is based on a similar process revolving around I_to_ [38].

Historically, ER was considered a benign finding on an ECG, but research now indicates that it can be a valuable predictor of malignant arrhythmia and SCD, particularly if present in the inferior or inferolateral leads on an ECG [39]. Furthermore, ER has consistently had a relatively high prevalence in the population, with studies finding that approximately 25% of males and 16% of females presented with the pattern in 1936, and between 3 and 24% still present with it now. However, this increased chance of presentation with ER does not imply an increased risk of malignant arrhythmia. The subgroups that are at a higher risk of malignant arrhythmia with ER are patients with structural heart disease and those of Asian ethnicity [40].

Antzelevitch et al., and Yakkali et al., described the presence of ER using the following criteria: A QRS notching/slur on the downslope of a prominent R wave (this must be above the isoelectric baseline) and a J-point greater than 0.1mV in two consecutive leads, excluding V1– V3 [39, 40]. There have been two descriptions of risk profiling based solely on the pattern of the ECG. The first described three core subtypes: low, medium, and high risk. Low risk demonstrated the ER pattern in the lateral leads only (type I); medium risk demonstrated an ER pattern in the inferior or inferolateral leads (type II), and high risk was associated with a widespread ER pattern (type III) [41].

The second proposal of risk stratification described either a benign or malignant ER pattern. Benign ER had a fast-sloping ST segment, whilst malignant ER had a horizontal or falling ST segment [40]. Other key determinants for risk stratification include a family history of SCD, cardiac arrest, syncope of severe criteria, associated pathologies such as BrS or fQRS, dynamic J wave changes, short-coupled premature ventricular contractions (PVC), and a J point amplitude greater than 0.2mV [40]. The practitioner should consider the appearance of the ER pattern in BrS, considering that it may not be spontaneous and only visible with class I antiarrhythmic drug induction, before disregarding the presence of early repolarisation in a patient.

### Inferolateral ER pattern

The first systematic investigation of inferolateral ER in BrS was performed by Sarkozy et al. [42]. The study focused on 280 patients with BrS, finding that 43 presented with an inferolateral ER pattern (15%). A study performed by Kawata et al., provided evidence of this increased risk of malignant arrhythmia when ER was present in the inferolateral ECG leads in BrS patients, focusing on 49 patients with type 1 BrS and a history of VF; they investigated the correlation between ER and recurrence of VF. In the study, three groups were identified: patients with persistent ER (30.6%), intermittent ER (32.7%), and no observable ER (36.7%). From this, it was found that patients with persistent ER had a 100% recurrence rate, intermittent ER carried a 75% recurrence rate, and no observable ER had a 44% recurrence rate. This provided evidence that the prevalence of ER in the inferolateral leads in patients with BrS was a significant predictor of malignant arrhythmia when either persistent or intermittent ER was present (HR 4.88, 95% confidence interval 2.02–12.7, *P* = 0.0004; and HR 2.50, 95% confidence interval 1.03–6.43, *P* = 0.043, respectively) [43].

This was also supported by a study by Tokioka et al., which included a larger sample of 246 patients with BrS. Out of the 246, 25 demonstrated an inferolateral ER pattern on their ECG, and 8 of these patients experienced either VF or SCD in the follow-up period. Episodes of VF/SCD were also observed more frequently in BrS patients with ER, than BrS patients without (*P* = 0.001 and *P* = 0.005, respectively). This supports the previous study that ER can be a significant predictor of malignant arrhythmia in BrS patients [44]. Importantly, this study also demonstrates that inferolateral ER is a powerful independent predictor with a HR of 6.029, 95% CI of 2.258 − 16.103, and a P value of < 0.001 [44]. It is important to note that this evidence does not directly translate to BrS under paediatric care. Lopez-Blazquez et al., performed a study at Great Ormand Street Hospital to evaluate traditional markers for risk stratification of malignant arrhythmia in BrS patients, including a cohort of 90 patients aged 18 or younger over a follow-up period between August 2003 and February 2019. From this cohort, 43 patients had BrS, the other 47 served as a control. Inferolateral ER was present in 28% of both the BrS and control cohorts. Out of this subgroup with inferolateral ER, only one was considered a high-risk phenotype, while 11 other patients were considered low-risk (*p* = 0.675). Furthermore, no significant arrhythmias were detected in the high-risk patient, although they experienced syncope [45]. The lack of correlation between inferolateral ER and MAEs displayed in this study highlights the need for further research to determine whether the traditional markers for risk stratification are genuinely suitable for paediatric patients with BrS, since there is currently only a handful of studies available on this.

### S Wave presence in lead I

BrS has various ECG manifestations, one of which may be the presence of an S wave in lead I. Lead I reflects conduction at the RVOT level and indicates a conduction delay, confirmed by insights from invasive epicardial mapping [46]. This is significant in the context of BrS, as abnormalities in epicardial action potentials are common due to the loss of function of sodium channels. This leads to changes in action potential heterogeneity in different segments of the right ventricle, most notably in the upper part of the RVOT, thereby providing a substrate for arrhythmogenesis [47].

The S wave in lead I has been known to increase the risk of SCD in BrS since 2016 [48]. The first investigation was conducted with a cohort of 347 patients, finding that the presence of an S wave in lead I was not only a predictor of VF but also the most potent marker present on an ECG when considered significant. The criteria for a significant S wave were defined as an amplitude equal to or greater than 0.1 mV or a duration equal to or greater than 40 ms. From the cohort, 32 (9.2%) individuals developed VF or suffered SCD, 39 (11.2%) experienced syncope, and the remaining 276 (79.5%) were asymptomatic. Among these groups, those who experienced VF/SCD were found to have S waves more commonly in lead I, with 96.9% of patients presenting with an S wave and 90.6% of those being classified as having a significant S wave. The S wave was also present on the ECGs of non-VF patients, however these were at a lower frequency [48]. This study amply demonstrates a new accurate marker for SCD risk assessment in BrS patients with sensitivities of 90.6%, 96.9%, and 96.9%, and specificities of 62.2%, 61.1%, and 69.5% for S-wave amplitude ≥ 0.1 mV, duration ≥ 40 ms, and amplitude duration area ≥ 1 mm², respectively.

Following the ECG findings, EPS were performed, confirming the mechanism behind this finding, with longer endocardial activation times found in the RVOT of patients with S waves compared to those without: 102.0 ± 41.2 ms vs. 51.5 ± 31.4 ms (*p* < 0.05), respectively. The anterolateral region of the RVOT demonstrated the most significant delays, with activation durations of 41.2 ± 24.3 ms (with S waves) compared to 8.4 ± 3.7 ms (without S waves) [48]. These findings proposed the presence of an S wave in lead I to be a significant marker of VF/SCD for the first time, and further confirmed in subsequent studies [49].

Another proposed risk marker for the risk stratification of VA in BrS is an SII > SIII pattern, where the S wave in lead II has a greater amplitude than in lead III. Both this pattern and a significant S wave in lead I were identified as significant independent predictors of VA (*P* = 0.006 and *P* = 0.025, respectively) [50]. This study suggested that the SII > SIII sign is a more prominent risk marker than a significant S wave based on amplitude (greater than 0.15), with SII > SIII having a higher odds ratio for developing VA [50]. This risk marker has also been shown to be relevant for paediatric population, with a study demonstrating among six BrS patients who had a prior arrhythmic event, and exhibited an S wave on the ECG had a 100% recurrence rate (HR: 3.1, 95% CI 1.04–9.30, *P* = 0.04) [51].

A limitation of this proposed risk factor is that it has not been reproducible in all broader studies focused on multiple variables. For example, Subramanian et al.’s., study found that the S wave amplitudes and durations were relatively similar in groups that experienced an arrhythmic event and those that did not. The mean S wave amplitude was 0.10mV in patients without a significant arrhythmic event and 0.11mV in those who did experience an event, while the mean S wave duration was 45ms and 48ms, respectively (*P* = 0.234 for amplitude and *P* = 0.351 for duration) [52].

### Spontaneous type 1 ECG pattern in leads other than the right precordial

Type 1 ECG pattern has been widely recognised as a critical marker for diagnosing BrS and assessing the risk of VAs and SCD. However, there has been increasing interest in whether similar Type 1 patterns observed in non-right precordial leads can provide additional diagnostic and prognostic value. Despite this, the precise diagnostic accuracy of these patterns in non-precordial leads remains undefined due to inconsistent reporting of sensitivity and specificity across studies [53, 54].

In cohorts involving 358 and 381 patients, Type 1 patterns detected in leads such as V1 and V2 were associated with HR of 6.3 (95% CI 1.4–28) and 10 (95% CI 1.8–54) at presentation, respectively, with follow-up HR of 3.1 (95% CI 1.3–7.2) and 5.3 (95% CI 1.6–17) [55–57]. Similarly, Rollin et al., reported that the detection of a spontaneous Type 1 pattern in at least one peripheral lead was associated with an increased risk of SCD and the need for ICD therapy in asymptomatic BrS patients [58]. Crea et al., further observed that ST depression in inferior leads was a common feature in patients with spontaneous Type 1 ECG patterns and was associated with an increased risk of VA [59]. Probst et al., demonstrated that spontaneous Type 1 ECG patterns in multiple leads, including non-right precordial leads, correlated with higher HRs for MAEs [60]. Interestingly, studies have also shown that patients with type 1 ECG patterns in peripheral leads often had SCN5A mutation, higher J-wave amplitudes in the precordial leads, and similar patterns in the peripheral leads [53]. Nevertheless, the diagnostic value of spontaneous Type 1 patterns when recorded outside the right precordial leads need to be investigated in large-scale studies.

### PR interval & QRS duration

The prognostic relevance of PR interval prolongation in BrS is underpinned by its association with mutations in the SCN5A gene, which encodes the cardiac sodium channel Nav1.5. These mutations disrupt sodium current conduction, leading to atrioventricular (AV) conduction delay and manifesting as a prolonged PR interval. However, SCN5A mutations have a wide phenotypic expression that extends beyond BrS, including atrial fibrillation (AF), sick sinus syndrome, Lenègre-Lev disease, and other forms of progressive cardiac conduction disease [61]. This broader spectrum of clinical manifestations highlights the systemic nature of sodium channel dysfunction and suggests that PR interval prolongation may represent more than just a BrS-specific risk marker, it may be indicative of widespread conduction system involvement beyond the RVOT.

Evidence supporting this perspective includes findings by Maury et al., who reported a 16.5–40% prevalence of first-degree AV block (PR ≥ 200 ms) in BrS patients significantly linked to adverse outcomes including SCD and appropriate ICD interventions (OR 2.41, *p* = 0.046) [37]. Similarly, Migliore et al., also showed that in patients with spontaneous type 1 Brugada ECG patterns, the presence of first-degree AVB was an independent predictor of MAEs and outperformed the type 1 ECG pattern itself in stratifying risk [62]. Meta-analytical evidence by Pranata et al., confirmed these associations, while gender-specific findings by Benito et al., revealed that prolonged PR intervals may carry stronger prognostic weight in female patients [3, 63]. Recent long-term multicentre data further reinforce the prognostic value of conduction abnormalities, showing that first-degree AV block was present in 17% of BrS patients receiving prophylactic ICDs, and significantly associated with late appropriate ICD therapies after generator replacement (log-rank test, *P* = 0.04). Notably, a subset of asymptomatic patients, traditionally considered low risk, experienced MAEs after generator replacement, particularly when baseline ECG revealed first-degree AV block. These findings highlight that even asymptomatic BrS patients with conduction abnormalities may harbour persistent arrhythmic risk, underscoring the importance of incorporating such markers into long-term risk stratification [64].

QRS duration, likewise, has proven to be a valuable metric in risk stratification. Rattanawong et al., highlighted in a systematic review that a wide QRS complex significantly increases the risk of MAEs [65]. Couto Pereira et al., further demonstrated that a QRS duration ≥ 119 ms was associated with a HR of 7.25 for MAEs [66]. Tse et al., applied automated ECG analysis techniques and found that prolonged QRS duration enhanced predictive accuracy for VA [67]. Similarly, Sieira et al., incorporated QRS duration into a multiparametric risk model, which successfully stratified patients by their arrhythmic risk [68]. Additional studies have supported the use of lead-specific measurements, such as QRS prolongation in leads V2 and V6, which were associated with symptomatic BrS and VF, as well as broad S waves ≥ 80 ms in lead V1, which showed good sensitivity and specificity for VF prediction [69–71].

Nevertheless, these findings must be interpreted with nuance. While PR interval prolongation and QRS duration are important indicators of electrophysiological disturbance, it should not be considered a definitive predictor of arrhythmia when viewed in isolation. Rather, it represents a non-specific manifestation of a broader pathophysiological substrate related to sodium channel dysfunction. Its clinical utility lies in its integration within a multimodal risk assessment strategy, as exemplified by the BRUGADA-RISK score proposed by Honarbakhsh et al., which incorporates ECG variables, clinical history, and other dynamic markers to improve predictive accuracy [72]. Therefore, PR interval prolongation should be seen not merely as a diagnostic marker, but as a signpost of more extensive sodium current impairment, underscoring the need for comprehensive risk stratification approaches that go beyond the traditional BrS criteria.

### The aVR sign

Building upon these conduction-related markers, attention has also turned to other ECG features that may improve predictive accuracy. One such marker is the aVR sign, characterised by an R-wave amplitude ≥ 0.3 mV or an R/Q ratio ≥ 0.75 in lead aVR [73]. Evidence suggests that the aVR sign is more prevalent in symptomatic individuals and those with SCN5A mutations, with studies reporting a specificity as high as 98.4% and HR for arrhythmic events ranging from 1.88 to 4.8 [74, 75]. As with PR interval and QRS duration, standardisation in measurement and broader validation are needed to fully embed the aVR sign into clinical practice. Nonetheless, its high specificity underscores its potential role in refining current risk stratification models for BrS.

A systematic analysis of thirteen studies involving 19,472 BrS patients indicated that the prevalence of the aVR sign varied significantly across populations, ranging from 10 to 38%. The aVR sign showed moderate sensitivity (62.5%) but notably high specificity (98.4%) for predicting arrhythmic events and SCD in BrS patients [76].

Several studies have reported varying effect estimates associating the aVR sign with adverse cardiac outcomes. Specifically, a prospective cohort study by Subramanian et al., reported a HR of 1.88 (95% CI: 1.21–15.67), suggesting significant predictive utility [74]. Ragab et al., reported an odds ratio of 4.8 (95% CI: 1.79–13.27), and Iqbal et al., reported a risk ratio of 2.00 (95% CI: 1.42–2.83), highlighting consistent associations with elevated arrhythmic risk [75–78].

Further investigations have indicated that the presence of the aVR sign correlates more strongly with certain clinical and genetic subgroups. For instance, Ragab et al. found the aVR sign to be more frequent among symptomatic patients [75]. Meanwhile, Rollin et al., observed that this ECG marker was prevalent among patients with SCN5A gene mutations, suggesting a genetic correlation [58].

Despite its predictive strengths, heterogeneity in definitions and measurement across studies complicates the standardised use of the aVR sign. Explicit definitions were employed in only a minority of studies, while others provided no clear criteria, limiting direct comparability and generalisability. Nevertheless, the presence of an R wave in lead aVR remains a valuable indicator of arrhythmic risk. Notably, a study involving 64 patients with ventricular tachycardia (VT) or frequent PVCs demonstrated that an R wave in aVR could localise arrhythmic origins to inferior, inferolateral, or apical ventricular regions [79]. This suggests that the aVR sign may not only reflect broader myocardial involvement but also serve as a non-invasive tool to identify the likely anatomical substrate of arrhythmia, thereby supporting its integration into comprehensive risk stratification frameworks for complex BrS phenotypes.

### T wave alternans

T-wave alternans (TWA) describes a pattern of beat-to-beat variability in the T-wave segment of the ECG, typically indicating instability in the cardiac repolarisation process. Initially observed by Lewis in the early 20th century, this phenomenon has since been recognised as a marker of electrical vulnerability within the myocardium [80]. At the electrophysiological level, TWA is linked to fluctuations in action potential duration (APD) and intracellular calcium cycling when the heart is paced at a consistent rate. In homogeneous tissue, this can present as spatially concordant alternans, where repolarisation alternates in a uniform pattern. However, in more complex or pathological conditions, spatially discordant alternans (SDA) can arise, where regions of the myocardium alternate out of sync. SDA, in particular, is pro-arrhythmic due to its association with heightened spatial dispersion of repolarisation, which facilitates unidirectional block and re-entry circuits [81–83].

In the context of BrS, TWA has gained increasing attention as a potential non-invasive marker for identifying high-risk individuals. While TWA on its own may offer limited specificity, its utility is significantly enhanced when incorporated into multiparametric models. For instance, Kawazoe et al., demonstrated that combining TWA with Tpeak–Tend (TpTe) dispersion and other ECG markers achieved a sensitivity of 97.1% for predicting VF, albeit with moderate specificity (63–65.7%) [84]. Similarly, Rattanawong et al., reported that the Predicting Arrhythmic event (PAT) score a composite model incorporating TWA yielded high diagnostic accuracy, with 95.5% sensitivity and 89.1% specificity for predicting initial MAEs [85].

Yoshioka et al., further supported TWA’s predictive capacity, revealing discriminant ratios of up to 93% when T-wave amplitude variability, a TWA proxy, was combined with LPs. Such findings underline TWA’s role in identifying high-risk BrS patients effectively, especially when utilised alongside additional ECG markers [86].

However, despite its excellent sensitivity, TWA’s predictive accuracy is notably enhanced when integrated into composite risk scores and advanced machine learning models. Lee et al., demonstrated that novel composite risk scores and machine learning techniques achieved superior overall predictive accuracy, with AUC values reaching up to 0.97. These approaches surpassed traditional risk markers, including isolated clinical history or ECG parameters alone, such as spontaneous type 1 ECG or fragmented QRS [77].

### Transmural dispersion of repolarisation

Transmural dispersion of repolarisation has been identified as a central mechanism in arrhythmogenesis among BrS patients. Several ECG-derived parameters such as the TpTe interval, TpTe dispersion, TpTe maximum value, TpTe/QT ratio, QTc prolongation, and QT dispersion have been explored for their ability to stratify arrhythmic risk and predict SCD. The TpTe interval, reflecting differences in repolarisation timing across the ventricular wall, is consistently prolonged in high-risk BrS patients and has been strongly linked with VF and other MAEs [87].

For instance, Maury et al. and Tronconi et al. demonstrated that TpTe ≥ 100 ms significantly increased the odds of SCD, atrial tachycardia, and appropriate ICD interventions [88, 89]. However, the utility of TpTe as a standalone risk marker remains contentious. Studies such as those by Mugnai et al., and Thapanasuta et al., reported no significant predictive association [90, 91]. Meta-analyses by Tse et al., and Pranata et al., support the prognostic value of TpTe and TpTe/QT ratios, yet underscore methodological inconsistencies, particularly wide variability in cut-off thresholds (ranging from 87 to ≥ 120 ms), differences in measurement technique, and the lack of prospective validation in diverse populations [92, 93].

Meanwhile, QTc prolongation and QT dispersion, which reflect global repolarisation heterogeneity have also demonstrated positive predictive values, particularly among symptomatic individuals [94]. Despite their promise, these parameters are again limited by a lack of standardised cut-offs, inconsistent reporting, and insufficient external validation. Consequently, their greatest clinical utility may lie in composite risk models, where they can enhance the accuracy of stratification when used alongside other ECG and clinical markers [92, 94].

### TZOU criteria

The Tzou criteria, originally developed to identify basal-lateral scar in patients with non-ischaemic cardiomyopathy (NICM) and VT have recently been explored for their prognostic utility in BrS. These ECG-based markers include a V1 R wave amplitude ≥ 0.15 mV, V6 S wave amplitude ≥ 0.15 mV, and a V6 S: R ratio ≥ 0.2. In NICM, these features were strongly predictive of epicardial or endocardial basal-lateral left ventricular scarring, which is often implicated in the arrhythmogenic substrate. In a two-phase study involving 25 NICM patients with VT and scar confirmed by detailed mapping, these criteria demonstrated 86% sensitivity and 88% specificity for identifying basal-lateral scar, independent of LV ejection fraction or QRS duration [95].

Given the presence of right ventricular conduction delay in BrS often producing an rS pattern in lead V1 and a prominent S wave in V6, investigators have hypothesised that the Tzou criteria may similarly reflect underlying substrate abnormalities predictive of VAs in this condition [50]. Early investigations suggested that BrS patients meeting the Tzou criteria exhibited a higher burden of symptomatic presentations and ventricular arrhythmias, supporting their potential role as independent risk markers. However, findings have been mixed across cohorts, with some studies failing to identify significant differences in Tzou criteria between symptomatic and asymptomatic patients or those with and without documented arrhythmic events [48, 50].

### Ambulatory electrocardiographic monitoring

Holter monitoring in BrS serves as a crucial tool for detecting dynamic arrhythmias and transient ECG changes not always visible on standard resting 12-lead ECGs. This continuous ambulatory ECG recording captures spontaneous VAs and variations in conduction, providing additional prognostic information beyond what is possible in brief clinical evaluations [66]. One of the findings on Holter monitoring in BrS is the presence of PVCs, particularly those originating from the RVOT. Holter monitoring enables the detection of PVCs and non-sustained VT, both of which have been associated with an increased risk of SCD [96].

In a prospective single-centre study of 117 BrS patients followed over 4.1 years, prolonged QRS duration and frequent PVCs were significantly associated with MAEs. Patients who experienced SCD or appropriate shocks for VA had longer baseline QRS durations and higher PVC counts on 24-hour Holter monitoring. A QRS duration of ≥ 119 ms and more than six PVCs per 24 h were associated with a 7-fold and 5-fold increased risk, respectively, highlighting their potential as non-invasive risk markers in BrS [66].

Additional studies have further demonstrated the prognostic significance of Holter monitoring in capturing spontaneous Type 1 ST-segment elevation. Extramiana et al., in a case-control study of 34 BrS patients undergoing 24-hour 12-lead ECG monitoring, found that a greater burden of spontaneous Type 1 ST-segment elevation in lead V2 was associated with the presence and severity of clinical symptoms [97]. Supporting this, a 2016 case study described a young patient who experienced VAs triggered by PVCs with short coupling intervals during periods of high vagal tone. Arrhythmic events occurred predominantly at night, with Holter recordings showing diminished ST-segment elevation during the day and enhanced expression at night [98]. These findings are consistent with earlier electrophysiological data showing that vagotonic agents can intensify ST elevation and increase arrhythmia inducibility, whereas sympathomimetic agents tend to have a suppressive effect [99–101].

More recently, Gray et al., conducted a cohort study involving 54 patients, including 32 with drug-induced BrS, and found that 34% exhibited spontaneous Type 1 ECG patterns during 24-hour Holter monitoring. A higher temporal burden of Type 1 ECG morphology was associated with increased cardiac event risk [102]. In line with this, Scrocco et al., reported that spontaneous Type 1 patterns were detected in up to 12% of individuals initially presenting with concealed BrS when monitored using 12-lead, 24-hour Holter ECG [55]. These findings underscore the value of Holter monitoring in revealing concealed but clinically relevant BrS patterns and guiding risk stratification.

Holter data also reveal important temporal variability in Brugada ECG patterns, with spontaneous fluctuations in ST-segment elevation magnitude and morphology. These dynamic changes often correspond to shifts in autonomic tone, fever, or medication effects, which can transiently heighten arrhythmogenic risk [87]. Nocturnal arrhythmias are of particular concern, as vagal predominance during sleep may exacerbate conduction delays and electrical heterogeneity, contributing to the observed clustering of BrS-related sudden deaths during rest or sleep.

Collectively, these findings highlight that 24-hour 12-lead Holter monitoring is a valuable diagnostic and prognostic modality in BrS. It allows for the detection of transient spontaneous Type 1 ECG patterns, ventricular ectopy, and arrhythmogenic triggers, all of which inform risk stratification. Future research should aim to refine Holter-derived metrics and integrate them with clinical, genetic, and invasive data to optimise personalised management strategies for patients with Brugada Syndrome.

### Exercise stress testing

Although traditionally utilised for the assessment of coronary artery disease, the exercise stress test has been explored as a potential tool in the risk stratification of patients with BrS. In the context of BrS, its value lies not in ischaemic detection but rather in the evaluation of dynamic ECG changes and arrhythmic susceptibility during periods of increased autonomic modulation, particularly vagal withdrawal and subsequent sympathetic activation [103].

The exercise stress test serves as a crucial tool for evaluating how the heart responds to physical stress. Patients with BrS frequently experience symptoms such as syncope, palpitations, and VAs. These symptoms tend to manifest during the early recovery period after exercise, when the heart rate is still transitioning back to its resting state. Notably, the exercise induced arrhythmias were thought to be linked to a vagal reaction, which increases parasympathetic tone during the recovery phase. This physiological response mirrors that of sleep, when the risk for SCD is heightened for BrS patients [103, 104]. Similarly, studies have shown that ECG changes are often evident post exercise, with one study demonstrating 57% of BrS patients exhibited significant ST-segment elevation immediately following exercise [104].

The diagnostic significance of BrS includes identifying patients with intermittent or concealed ECG patterns influenced by modulating factors such as increased vagal tone, decreased adrenergic tone during recovery, and elevated body temperature during exercise. The risk of these changes has been recently explored in several studies. Amin et al., observed that exercise exacerbates the ECG phenotype in BrS, with SCN5A mutations linked to slower conduction at higher heart rates [105]. They also found that increased ST-segment elevation during early recovery from exercise stress testing strongly predicted spontaneous VF. Subramanian et al., studied 75 asymptomatic BrS patients who underwent treadmill testing and were followed for six years, identifying three independent predictors of MAEs: an increase in S-wave upslope > 30% at peak exercise in precordial leads, J-point elevation of ≥ 2 mm in lead aVR during late recovery, and a heart rate fall of < 40% from maximum in late recovery. Among those with an unfavourable result, 80% exhibited all three predictors, leading the authors to conclude that exercise stress testing is a simple and effective tool for risk stratification in asymptomatic patients with type 1 BrS pattern [74]. Similarly, Makimoto et al., reported that one-third of BrS patients undergoing exercise stress testing exhibited augmented ST-segment elevation in early recovery, an independent predictor of VF during follow-up. This augmentation, defined as an increase of > 0.05 mV in leads V1-V3 within 1–4 min post-exercise compared to resting ECG, was associated with higher heart rate recovery, suggesting a vagally mediated response [106]. More recently, Morita et al. studied 307 BrS patients, 73% of whom were asymptomatic, and found that ventricular extrasystoles occurring 1.5–3 min post-exercise were associated with VF during a follow-up period of 92 ± 68 months [107].

Collectively, these findings indicate that exercise stress testing not only offers important insights into the heart’s functional response to stress but also serves as a useful predictor of arrhythmic risk. Nevertheless, further research is needed to confirm if exercise stress testing can aid in clinical decision-making, particularly in determining the need for implantable devices to help prevent life-threatening arrhythmias in BrS patients.

### Cardiac imaging

Echocardiography remains one of the most accessible imaging tools for initial cardiac evaluation in BrS. Traditionally used to exclude overt structural cardiomyopathies, the advances in speckle-tracking (STE) and three-dimensional (3D) echocardiography have revealed consistent subclinical abnormalities in BrS patients. While BrS has long been considered a purely electrical disease, accumulating imaging evidence challenges this notion, highlighting subtle structural and functional alterations, particularly involving the RV.

Strain imaging, including STE and cardiac magnetic resonance (CMR) feature tracking, has uncovered impairments in myocardial deformation. Multiple studies have found significantly reduced RV strain parameters in BrS patients [108, 109]. In a key prospective study, Mitroi et al., demonstrated that RV strain markers, specifically a myocardial performance index (RIMP > 0.50), reduced RVOT strain (< 16.2%), and increased mechanical dispersion (RVMDm > 42 ms) were predictive of arrhythmic events over a 7-year period [109]. Hohneck et al., similarly reported impaired RV global circumferential strain and enlarged RVOT dimensions in spontaneous BrS cases, which correlated with MAEs [108].

Emerging evidence also suggests that myocardial deformation imaging of the LV may reveal an underappreciated arrhythmogenic substrate. In a cohort study involving BrS patients with prior electroanatomic mapping, LV mechanical dispersion (MD) ≥ 40 ms was associated with significantly larger areas of unipolar low-voltage (< 5.5 mV), suggesting greater substrate involvement [110]. Although RV deformation parameters did not correlate with electroanatomic or genetic findings in that study, other research, including work by Kukavica et al., and Scheirlynck et al., has identified RV dyssynchrony and mechanical clustering abnormalities using STE that were predictive of VA risk [109, 111, 112]. Pappone et al., extended these insights using 3D echocardiography, demonstrating that electromechanical substrate abnormalities strongly predicted VT and VF events [113].

In addition to deformation analysis, imaging has identified abnormalities in contraction timing and ventricular synchrony. Van Malderen et al., reported that BrS patients with prior syncope or VA exhibited prolonged RV and interventricular ejection delays, with a delay of > 40 ms in spontaneous type 1 ECG cases predicting arrhythmic events with 85.7% sensitivity and 93.7% specificity [114]. Trevisan et al., further observed dynamic RVOT contraction delay coinciding with spontaneous ECG pattern emergence [115].

Cardiac MRI offers high-resolution anatomical and tissue characterisation, particularly useful for assessing arrhythmogenic substrates. CMR studies have revealed reduced RV ejection fraction and structural changes in the RVOT and anterior wall [108, 116–118]. Hohneck et al., found abnormal RV strain in up to 81% of BrS patients, with strong associations to MAE [108]. Rudic et al., and Massara et al., observed that RV dilation, reduced RV ejection fraction, and strain abnormalities correlated with SCN5A mutation status and syncope, with the latter proposing a multiparametric scoring system for improved risk prediction [53, 119].

Tissue characterisation by CMR has produced mixed findings. While late gadolinium enhancement (LGE) is uncommon (~ 7–8%), when present, it strongly predicts arrhythmic risk. Murakami et al., showed that LGE correlated with VF inducibility, and Isbister et al., documented longitudinal deterioration in RV function and fibrosis [120, 121]. Pappone et al., demonstrated that structural MRI abnormalities predicted VT/VF events even in phenotypically silent BrS cases. Fat infiltration, typically a hallmark of ARVC, has also been inconsistently reported [122]. Papavassiliu et al., observed it in 15% of BrS patients, whereas Catalano et al., found none, likely reflecting limitations in spatial resolution, especially in the thin RV wall [116, 117]. Notably, post-mortem histology suggests a higher prevalence of fibrosis and fatty replacement than is detectable by in vivo CMR [118].

Atrial involvement is an emerging field of interest. While early studies reported no atrial size differences between BrS patients and controls, Bastiaenen et al., observed increased right atrial area in SCN5A-positive individuals [117, 118]. More recently, Toh et al., found enlarged left atrial diameter and volume in BrS patients with arrhythmias or SCN5A mutations, suggesting a possible broader electrical or structural atrial pathology [123].

Computed tomography (CT), although less routinely employed, has proven valuable in delineating RVOT morphology and assessing epicardial adipose tissue (EAT). Takagi et al., used electron-beam CT to identify focal RVOT morphological changes linked to PVCs [124]. Yonezu et al., showed that greater EAT volume and lower CT attenuation were strongly associated with VF incidence, suggesting a modulatory role for EAT in arrhythmogenesis [125].

A 2023 systematic review by De Raffele et al., synthesised echocardiographic and CMR findings, concluding that structural abnormalities, once thought epiphenomenal, play a substantive role in BrS pathophysiology and risk prediction. While variability in imaging modalities and diagnostic thresholds remains a challenge, the literature strongly supports incorporating imaging into BrS evaluation [54].

**Fig. 4:**
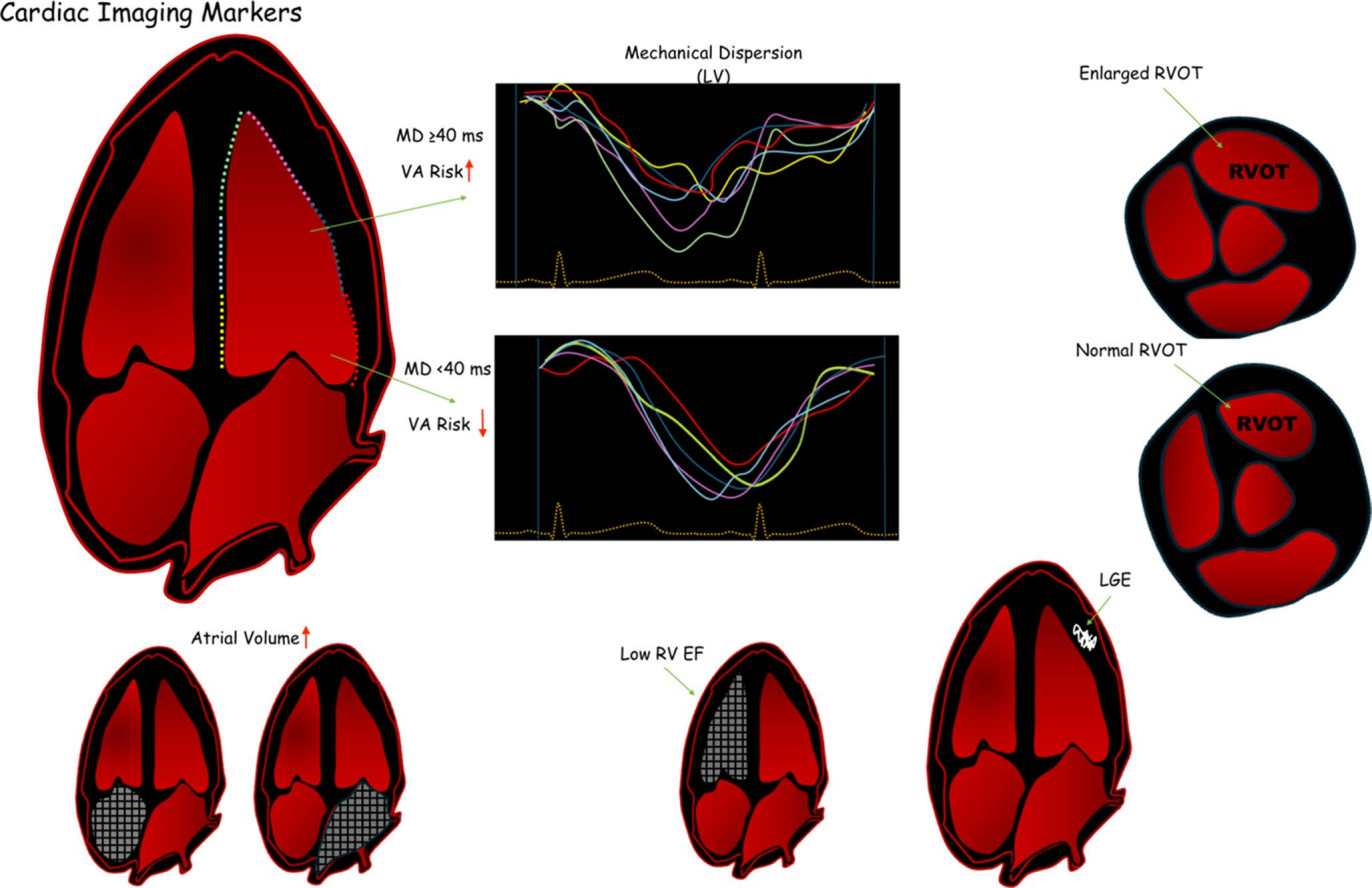
Examples of cardiac Imaging risk markers

Taken together, these findings challenge the traditional paradigm of BrS as a structurally normal heart disease. Instead, advanced imaging reveals subtle yet clinically significant abnormalities in ventricular mechanics, synchrony, and tissue structure, particularly in high-risk groups such as SCN5A mutation carriers and spontaneous type 1 ECG phenotypes. Each imaging modality contributes unique insights: echocardiography excels in dynamic and mechanical assessment; CMR provides superior tissue characterisation and functional analysis; and CT offers detailed anatomical mapping of the RVOT and epicardial structures (Fig. 4). When integrated with genetic, electrocardiographic, and electrophysiological data, cardiac imaging enhances arrhythmic risk stratification and supports a more holistic approach to BrS diagnosis and management.

### Electrophysiology study

Over the past decade, epicardial substrate mapping, particularly in the RVOT has provided increasing insight into the pathophysiological basis of BrS and its association with SCD. Pappone et al., identified significant mechanical and electrical abnormalities in the RV, revealing that electroanatomic mapping could localise a larger arrhythmogenic substrate associated with type 1 BrS. Ablation of these regions successfully eliminated both BrS ECG patterns and underlying mechanical disturbances [113]. Similarly, Ciconte et al. demonstrated that BrS patients exhibit an enlarged epicardial substrate, which plays a critical role in arrhythmogenesis [7].

A systematic review and meta-analysis evaluated the prognostic utility of electrophysiological study (EPS) in asymptomatic BrS patients without prior aborted SCD or fatal arrhythmic events. Nineteen studies including 6,218 patients were analysed, with 68.6% undergoing EPS. The findings confirmed that a positive EPS was significantly associated with a higher risk of future arrhythmic events (RR 1.74), and this association remained robust even in those without previous major events (RR 1.60), supporting the selective use of EPS in risk stratification and ICD decision-making for asymptomatic individuals [126]. However, a large multicentre registry of 165 asymptomatic patients with spontaneous type 1 ECG patterns undergoing EPS revealed a very low annual event rate of 0.15%, with no significant difference in MAE rates between inducible and non-inducible patients (0.54% vs. 0.06%, *P* = 0.16). Interestingly, markers such as first-degree AV block, prolonged QRS duration, and SCN5A positivity were more common in those who experienced MAEs, suggesting these baseline features may be of greater prognostic value than EPS alone. These data caution against using EPS as a sole determinant for ICD implantation in asymptomatic patients and advocate instead for a multiparametric approach [127].

Recent findings by Nagase et al., further reinforce the relevance of epicardial mapping in BrS. At baseline, patients with BrS demonstrated significantly higher J-wave amplitudes (≥ 0.42 mV) with bipolar delayed potentials, that yielded 99.1% sensitivity and 100% specificity for BrS diagnosis. In addition, a steep repolarisation gradient was observed in both ventricles, strongly correlating with VA risk [128].

A prospective registry study further investigated the prognostic significance of substrate size and the therapeutic impact of epicardial radiofrequency ablation (RFA) in symptomatic BrS patients. Among 257 individuals with ICDs, 206 underwent RFA. During the pre-ablation phase, independent risk factors for VF included a larger substrate size (HR 1.13), prior aborted cardiac arrest (HR 2.98), and SCN5A variants (HR 2.22). Over a median follow-up of 40 months, RFA significantly reduced VF recurrence with no major procedural complications, affirming both its safety and efficacy in high-risk patients [129].

### Risk scoring models

To enhance risk stratification in BrS, several scoring systems have been developed, including the Shanghai Score System, Sieira score, and the BRUGADA-RISK model. The Shanghai system, initially designed for diagnosis, incorporates ECG patterns, clinical history, family history, and genetics but shows limited specificity, with reported AUC values between 0.63 and 0.73. The Sieira score, focusing on syncope, spontaneous type 1 ECG, and family history, demonstrates better performance, with AUCs ranging from 0.71 to 0.82 [130]. The BRUGADA-RISK model adds spontaneous type 1 ECG, peripheral lead involvement, early repolarisation, and arrhythmic syncope, achieving a sensitivity of 71.2%, specificity of 80.2%, and an AUC of 0.83 for five-year arrhythmic event prediction [34, 73, 131].

A recent study developed a novel risk prediction tool called the PAT score, based on a systematic review and pooled analysis of 67 studies involving 7,358 patients worldwide. The PAT score incorporates 15 significant risk factors and was validated internally in 105 Asian patients and externally in 164 multiracial patients with BrS. Compared to existing scores; BRUGADA-RISK, Shanghai, and Sieira, the PAT score showed significantly superior predictive accuracy, with an external validation ROC of 0.9671 versus 0.7210–0.8174 for others. A PAT score ≥ 10 predicted first MAE with 95.5% sensitivity and 89.1% specificity. While prediction of recurrent MAE showed lower sensitivity (15.4%), specificity remained high (93.3%) [86].

This was further confirmed by Balla et al.’s., study which compared the predictive accuracy of four BrS risk scores; BRUGADA-RISK, Shanghai, Sieira, and PAT, in a real-world European cohort of 154 patients (mean age 51.3 years). MAEs included arrhythmia-related syncope or sudden cardiac arrest. The PAT score outperformed all others, showing the highest sensitivity (92.1%), specificity (93.1%), positive predictive value (83.3%), and negative predictive value (97.3%). It also had the greatest discrimination with an AUC of 0.97 (95% CI: 0.95–0.99) [132].

Given the heterogeneity and variable predictive performance of traditional risk stratification methods, recent advances have emphasised the application of machine learning to enhance predictive accuracy. A large retrospective cohort study of 548 Asian BrS patients developed and compared multiple machine learning models, including random survival forest, gradient boosting classifiers, and others. In the overall cohort, the Sieira score remained robust (AUC 0.806), but a novel score incorporating Sieira plus additional variables improved discrimination (AUC 0.855). A simple non-invasive clinical and ECG variable-based score showed comparable performance (AUC 0.784), which significantly improved with random survival forest machine learning methods (AUC 0.942). Among intermediate-risk patients (*n* = 274), a gradient boosting classifier demonstrated the best predictive accuracy (AUC 0.814) [78].

Ishida et al.’s., study also aimed to develop a risk prediction model for BrS using machine learning in a cohort of 234 Japanese patients. Clinical and ECG features, including syncope history, various interval measurements, and ECG patterns were analysed. Existing scores like BRUGADA-RISK and PAT showed modest predictive accuracy with AUCs of 0.57 and 0.59, respectively. Among the machine learning models tested, the support vector machine achieved the highest accuracy. A simplified support vector machine model using seven key features (r-J interval in V1, syncope history, fragmented QRS, early repolarisation presence, T-peak-to-T-end interval, QRS duration in V6, and age) yielded a significantly improved AUC of 0.77 [133]. The findings suggest that machine-based models, especially streamlined ones, may enhance risk stratification for MAEs in BrS patients.

### Future perspectives: computational models in Brugada Syndrome risk stratification

Artificial intelligence (AI) represents a rapidly evolving paradigm in the risk stratification of BrS, with the potential to enhance predictive performance beyond traditional scoring systems by extracting latent features from high-dimensional ECG data. Recent studies employing convolutional neural networks (CNNs) and classical machine learning algorithms, such as boosted decision trees (BDT), multi-layer perceptrons (MLP), and support vector machines (SVM) have demonstrated the capacity of AI to identify subtle electrophysiological signatures predictive of MAEs.

For instance, Nakamura et al., developed a CNN-based model trained on 2,053 ECG recordings from 157 BrS patients, achieving an AUROC of 0.80–0.81 for prior or future VF episodes. The model exhibited a notably high negative predictive value (0.94 ± 0.11), underscoring its utility in safely excluding high-risk individuals, although positive predictive value remained moderate (0.44 ± 0.29), reflecting the ongoing challenge in accurately identifying true positives [134]. Similarly, Randazzo et al., applied machine learning classifiers on manually extracted ECG features in a cohort of 209 BrS patients, with BDT attaining the highest F1-score (0.67) and consistent findings of high negative but limited positive predictive values [134, 135].

These findings highlight AI’s potential in augmenting BrS risk stratification, particularly in improving sensitivity and negative predictive capacity. However, current limitations include modest positive predictive performance, constrained sample sizes, and limited ethnic diversity within study populations. Additionally, issues related to data heterogeneity, lack of model transparency, and standardisation across centres represent barriers to widespread clinical translation.

Future research should focus on large, multi-centre studies with diverse patient groups to test AI models across different populations. Developing AI tools that are more interpretable and combining them with established clinical and ECG risk markers will be important to support clinical adoption. Ultimately, AI has strong potential to improve personalised risk prediction and help guide treatment decisions in Brugada Syndrome.

## Conclusion

BrS remains a diagnostically and prognostically challenging condition, particularly in asymptomatic individuals or those with concealed ECG patterns. While spontaneous Type 1 ECG and history of arrhythmic events remain cornerstone indicators, they fail to identify a significant proportion of patients at risk of SCD. This review highlights the growing body of evidence supporting a multiparametric approach to risk stratification, incorporating advanced electrocardiographic indices, signal-averaged ECG, Holter monitoring, cardiac imaging, and electrophysiological studies. ECG markers such as fragmented QRS, aVR sign, and T-wave alternans, alongside imaging indicators of RV strain and RVOT mechanical dispersion, challenge the historic paradigm of BrS as a purely electrical disease and point toward a structural-electrical interplay in arrhythmogenesis.

Furthermore, the emergence of risk scores like the PAT model, and machine learning tools trained on ECG and clinical data, offer promising precision stratification, particularly in intermediate-risk and asymptomatic cohorts where traditional models fall short. Integration of artificial intelligence into clinical workflows may enhance predictive accuracy and improve individualised care.

Ultimately, optimal management of BrS requires a tailored approach, balancing the benefits and risks of interventions such as ICD implantation. Future research should prioritise large, prospective, multi-ethnic cohort studies to validate emerging markers and integrate them into universally accepted stratification frameworks. As our understanding of BrS deepens, precision medicine will become increasingly attainable, enabling safer, more effective prevention of life-threatening arrhythmias in this complex syndrome.

## Electronic Supplementary Material

Below is the link to the electronic supplementary material.

Supplementary Material 1
