# Supplementary figures and images for: Beyond the type 1 pattern: comprehensive risk stratification in Brugada syndrome
Source: J Interv Card Electrophysiol. 2025 Aug 6;68(8):1771–90. doi: 10.1007/s10840-025-02101-z (PMC12476449; doi:10.1007/s10840-025-02101-z)

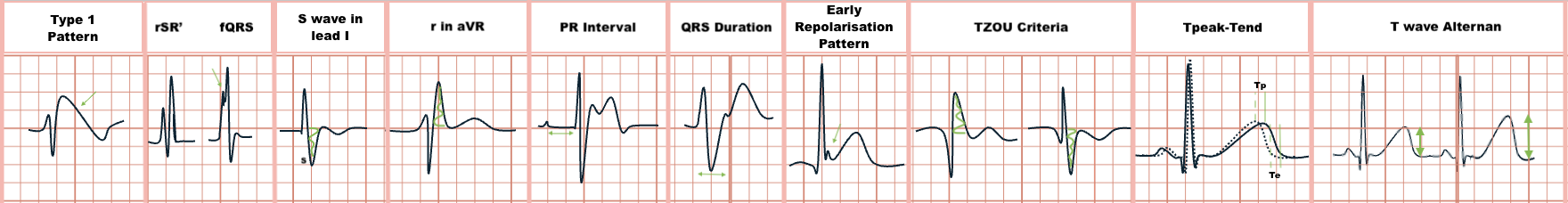
BRS ECG Risk Markers

Supplement: Supplementary file 1 — Supplementary Material 1 [file 10840_2025_2101_MOESM1_ESM.docx]
